# What do we need kids for? Childbearing motivations, personal values, and socio-demographic differences

**DOI:** 10.3389/fpsyg.2025.1612384

**Published:** 2025-09-03

**Authors:** Eugene Tartakovsky, Mor Mizrahi

**Affiliations:** The School of Social Work, Tel Aviv University, Tel Aviv, Israel

**Keywords:** childbearing motivations, general and context-specific motivations, personal values, preconception period, socio-demographic differences in values and childbearing motivations

## Abstract

**Introduction:**

In this study, we propose a new conceptualization of childbearing motivations and examine their links with personal values and socio-demographic variables during the preconception period.

**Method:**

To test our model, we conducted a cross-sectional study using a stratified sample of young Israeli Jews without children (aged 18–35, *n* = 1,122).

**Results and discussion:**

We found that childbearing motivations form four clusters, reflecting different goals people strive to achieve through childbirth. We referred to these clusters as life enrichment, authority, preservation, and perpetuity. The four clusters formed a two-dimensional circumplex paralleling the structure of values. The pattern of connections between childbearing motivations and personal values corroborated the existence of a contextualization mechanism linking general and specific motivations. In addition, the indirect effects of socio-demographic variables on childbearing motivations through personal values showed that differences in childbearing motivations across socio-demographic groups may be partly attributed to variations in general motivational goals that characterize the groups.

## Introduction

Childbearing is considered a universal human desire (Jenkins, 2020). However, studies on the motivational aspects of childbearing are surprisingly rare. Thus, we do not know enough about the motivations for having children and interpersonal differences in this regard. In this study, we propose a new conceptualization of childbearing motivations and examine their links with personal values and socio-demographic variables during the preconception period. We base our research on the theory of human values (Schwartz, 2017), the most comprehensive and empirically sound theory of motivations. Following the idea of “child’s values” (Gormly et al., 1987; Miller, 1994), we assume that people strive to attain various personal goals through childbearing. We further assume that individuals’ childbearing motivations are derived from their general motivational goals, as expressed in their values. Finally, we assume that group differences in values partially explain socio-demographic differences in childbearing motivations.

To test our theoretical model, we conducted a cross-sectional quantitative study in Israel, using a stratified sample of young Jews without children aged 18–35, *n* = 1,122. Studying childbearing motivations in Israel is interesting because it occupies a special place among other highly developed countries in terms of childbirth. First, Israel has the highest birthrate among developed countries (2.94). Similarly to most other developed countries, the birthrate in Israel is decreasing; however, it remains much higher than the “replacement level” (2.1 children per woman) in all socio-demographic groups in the country (Birenbaum-Carmeli, 2021). Childlessness rate in Israel is very low (6.4% among Jews, according to Weinreb et al., 2018), with only about 4% of young Israelis saying they do not want children (Tartakovsky and Mizrahi, 2025). Second, despite huge fertility differences across religious and ethnic groups in Israel, varying from 2.8 among non-Orthodox Jews to 6.8 among ultra-Orthodox Jews, strong pronatalist norms exist in all socio-demographic groups (Birenbaum-Carmeli, 2021; Weinreb et al., 2018). Third, almost all childbirths in Israel happen among married or cohabiting women, with only about 10%, mostly older women, giving birth out of wedlock (Weinreb et al., 2018). Thus, childbirth in Israel is tightly linked to the institute of marriage. Fourth, the high birthrate in Israel is promoted by the state using the pronatalist ideology, providing free IVF treatments, and maintaining a high level of free perinatal medical care (Granek and Nakash, 2017; Lavee and Katz, 2003; Waldman, 2006; Weinreb et al., 2018). At the same time, the parental leave in Israel is short (about 14 weeks), and child subsidies are small (Weinreb et al., 2018). Finally, the high birthrate in Israel has significant political implications, particularly considering the Arab-Jewish conflict and the tension between ultra-Orthodox and non-Orthodox Jews, and, therefore, it is a topic of heated political debates (Bartal, 2010; DellaPergola, 2003; Granek and Nakash, 2017).

### Conceptualization of childbearing motivations

Childbearing motivations answer the questions, “Why do I want a child? What are my goals in bringing a child into the world?” The conceptualization of childbearing motivations is based on the idea of “child’s values,” which refers to the potential benefits parents gain from having a child (Gormly et al., 1987; Guedes et al., 2015; Hofer et al., 2018; Matias and Fontaine, 2013; Miller, 1994). The motivations discovered in previous studies include status, novelty and fun, morality, creativity, accomplishment, influence, social comparison, economic utility, duty toward society, and continuity (Gormly et al., 1987; Guedes et al., 2015; Miller, 1994). However, these childbearing motivations have not been grounded in a psychological theory they are not content exhaustive (Gormly et al., 1987; Miller, 1994; Miller et al., 2004). In addition, previous studies have not investigated the motivational structure of childbearing motivations, i.e., their conflicts and compatibilities.

### Psychological and socio-demographic factors related to childbearing

We did not find any study investigating psychological variables affecting childbearing motivations. However, several studies examined the effects of psychological variables on childbearing intentions, desires, and attitudes (regarding having children, the number of children, and the optimal age of having the first child). We used the results of these studies to formulate our hypotheses on the childbearing motivational structure.

One line of research connects childbearing to religiosity because religion provides ideological incentives to bear children, regulates reproductive behavior, and promotes fertility (Inglehart, 2021; Jenkins, 2020). Empirical studies have found that religiosity and the traditional way of life are positively associated with childbearing attitudes and intentions (Gubernskaya, 2010; Hashemzadeh et al., 2021; Ranjbar et al., 2024; Tartakovsky and Mizrahi, 2025; Trepczyk and Szablewska, 2024).

In addition to fulfilling religious obligations, researchers have claimed that childbearing plays other important roles for the smooth functioning of the community, including providing intergenerational continuity, symbolic personal immortality, and social support for older parents (Barber, 2001; Groat et al., 1997; Inglehart, 2021). Empirical studies have demonstrated that prosocial attitudes and behavior are associated with having more children (Eriksson et al., 2020). Thus, previous studies have shown that childbearing serves not only to attain religious, but also other collectivistic goals (Inglehart, 2021; Ren et al., 2024).

Most researchers have assumed that individualistic motivations harm childbearing (Gubernskaya, 2010; Inglehart, 2021; Jenkins, 2020; Ranjbar et al., 2024; Ren et al., 2024). They claimed that individuals committed to autonomy, self-expression, self-realization, control over their bodies, sexuality, and intimate relationships are less inclined to childbearing. Indeed, socio-demographic variables associated with individualism and personal autonomy, such as the importance of obtaining an education and developing a career, have been linked to delays in childbirth and less positive attitudes toward childbearing (Barber, 2001; Hashemzadeh et al., 2021; Ranjbar et al., 2024; Trepczyk and Szablewska, 2024). However, other researchers have assumed that some individualistic motivations, especially related to self-development and joy of childcare, may promote childbearing (Nachoum et al., 2021; Hashemzadeh et al., 2021). Still, all researchers agree that collectivistic and individualistic motivations for childbearing contradict each other (Inglehart, 2021; Jenkins, 2020).

Another line of research on childbearing intentions and desires emerged from personality studies. Thus, one study found that high scores on the affiliation trait predicted stronger childbearing intentions (Miller, 1994). Another study found that agreeableness, nurturance, warmth, femininity, and empathic concern were associated with a stronger desire to have children (Buckels et al., 2015). These studies indicate the existence of childbearing motivations related to caring for others (Miller, 1994; Mynarska and Rytel, 2022).

We found the only study investigating gender differences in childbearing motivations (Mynarska and Rytel, 2022). In this study, men reported stronger motivations related to the joy of childbearing and traditional parenthood motivations, while women reported a stronger connectedness motivation. Education, career, economic independence, and hedonistic behavior (e.g., luxury spending) have been found to contradict childbearing in both genders (Barber, 2001; Groat et al., 1997; Trepczyk and Szablewska, 2024). However, these factors had a stronger impact on childbearing attitudes among women than men (Delbaere et al., 2020; Hashemzadeh et al., 2021; Trepczyk and Szablewska, 2024). The gender differences in childbearing intentions have been attributed to differences in values and behavioral norms among men and women (Mynarska and Rytel, 2022). In addition, the gender differences in childbearing intentions have been attributed to a stronger effect of childbirth on women’s education and career compared to men (Ranjbar et al., 2024; Trepczyk and Szablewska, 2024).

### Theory of human values

The proposed study is based on Schwartz’s theory of values, which defines values as desirable, trans-situational goals that guide people’s lives (Schwartz, 2017). In its latest formulation (Schwartz et al., 2012), the theory specifies a comprehensive set of 12 motivationally distinct basic values (some further divided into lower-level components): power (dominance and resources), achievement, hedonism, stimulation, self-direction (thoughts and actions), universalism (nature, tolerance, and concern), benevolence (care and dependability), humility, conformity (rules and interpersonal), tradition, security (personal and social), and face. In addition, the theory assumes the existence of dynamic relations between values: the pursuit of each value may conflict or be congruent with the pursuit of other values.

The conflicts and congruities among basic values yield an integrated structure of four higher-order value types arrayed along two dimensions: self-enhancement vs. self-transcendence and openness to change vs. conservation. Self-enhancement encompasses achievement and power values, emphasizing the pursuit of self-interest through demonstrating competence and attaining social status and dominance over others. Thus, self-enhancement values reflect the goals of self-promotion and self-empowerment. Self-transcendence includes the values of universalism and benevolence, emphasizing concern for the welfare and interests of others. Thus, self-transcendence values reflect the goals of care for others and promoting their interests, contradicting the self-enhancement values. Openness to change encompasses the values of self-direction and stimulation, emphasizing independent action, thought, and feeling, as well as a willingness to engage in new experiences. Thus, openness to change values reflect the goals of seeking independence and excitement. Conservation includes security, conformity, and tradition, emphasizing self-restriction, order, safety, and stability. Thus, conservation values reflect the goals of preserving the status quo, opposing openness to change values.

### The present study

The primary objective of this study was to examine the structure of childbearing motivations, focusing on their complementarities and conflicts. The second goal was to explore links between personal value preferences and childbearing motivations and to reveal the connection between general and specific motivational goals. Third, we aimed to test a theoretical model that claims socio-demographic variables are indirectly connected to context-specific motivational goals (childbearing motivations) through their relationship with general motivational goals (personal values). Thus, we aimed to demonstrate that socio-demographic differences in childbearing motivations result (at least partly) from the socio-demographic differences in value preferences.

Following previous studies on context-specific motivations and values (Czyżkowska and Cieciuch, 2022; Tartakovsky, 2023a, 2023b, 2025; Tartakovsky and Mizrahi, 2025), we assumed that the content and structure of childbearing motivations parallel those of human values and hypothesized that basic childbearing motivations form a circumplex with four clusters, similar to values (H1). We named these clusters life enrichment, authority, preservation, and perpetuity. *The life enrichment cluster* encompasses the following childbearing motivations: psychological growth, mastering parenting challenges, and enjoying the child; it is linked to openness to change values. *The authority cluster* encompasses proving oneself, controlling others, and social recognition, and is related to self-enhancement values. *The preservation cluster* encompasses strengthening the in-group, ensuring future support, fulfilling religious obligations, continuity, and yielding to social pressure, and it is related to conservation values. Finally, *the perpetuity cluster* encompasses motivations related to nurturance and contribution to humankind, which are related to self-transcendence values. We assumed that the order of childbearing motivations in the circumplex follows the rules of compatibility and conflict, such that motivations derived from opposing higher-order values are conflictual and are located on opposite sides of the circle. Thus, we hypothesize that the life enrichment cluster contradicts the preservation cluster, and perpetuity contradicts the authority cluster (H2).

We hypothesized that socio-demographic variables indirectly affect childbearing motivations through their connection to personal values. Age is associated with higher preferences for self-transcendence and conservation values (Schwartz et al., 2012). Therefore, we expect it to be associated with perpetuity and preservation motivations (H3). Being female is associated with higher preferences for self-transcendence and conservation values (Schwartz and Rubel, 2005); therefore, we expect women to have stronger perpetuity and preservation childbearing motivation (H4). Religiosity is associated with a higher preference for conservation values (Schwartz et al., 2012); therefore, we expect it to be associated with a higher preference for preservation and a lower preference for life enrichment motivations (H5). Education is associated with a higher preference for openness to change values (Schwartz et al., 2012); therefore, we expect it to be related to the life enrichment motivation (H6). Finally, income is associated with a higher preference for self-enhancement values (Schwartz et al., 2012). Therefore, it should be related to the authority childbearing motivation (H7).

## Methods

### Participants

The study used a stratified sample of young Jewish Israelis without children (n = 1,122, 47% male). We did not include Palestinian Israelis in the study because this group has distinctive fertility patterns, family structure, and value preferences (Lavee and Katz, 2003; Smooha, 2019; Tartakovsky, 2023a, 2024, 2025). The sample mean age was 26.2 (*SD* = 4.62, range = 18–35). 64% of the participants had a post-secondary education. 37% had salaries lower than the country’s minimum, 36% between the minimum and the average, 17% about the average, and only 10% of the participants had salaries higher than the country’s average. Forty-five percent of the participants identified themselves as secular (they did not follow any religious practices), 32% identified as traditional (they followed some religious practices), and 23% identified as religious (they strictly followed religious practices).

### Procedure

The Tel Aviv University Review Board approved the study. Two research companies conducted the survey under the supervision of the researchers in September 2023. The sample was drawn from the companies’ panels and stratified by gender, religiosity, and education. Jewish Israeli citizens aged 18–35 without children were invited to participate in the study. Participation in the study was voluntary and anonymous. All participants signed a written informed consent form and received a standard compensation of about $5. The study was conducted in Hebrew. The questionnaires were distributed using Qualtrix.

### Instruments

#### Childbearing motivations

The Childbearing Motivations Scale was developed for this study. The scale consists of 70 items adopted from previous studies (Gauthier et al., 2007; Gormly et al., 1987; Miller, 1994) and collected through interviews conducted with 51 Jewish Israelis (aged 20–40 years, 31 of whom did not have children), who were asked about their motivations for having children. Two researchers classified all items according to their meaning in terms of general motivational goals reflected in values (Schwartz et al., 2012) and organized them into 13 childbearing motivations, each related to a specific value (4–6 items per scale). Respondents indicated how important each childbearing motivation is to them on a 6-point scale, from 1 – *not important at all* to 6 – *very important*. Table 1 lists values, their corresponding motivational goals, childbearing motivations with example items, and the internal reliability of childbearing motivations scales. Correcting for individual differences in using the response scale, participants’ responses were centered on their mean for all childbearing motivations.

**Table 1:** Values and childbearing motivations: the hypothesized structure.

Values and their motivational goals	Childbearing motivations and motivational clusters, example items, number of items, and Cronbach’s alphas
***Openness to change higher-order values***	***Life enrichment***
*Self-direction*: Freedom to cultivate one’s ideas and abilities and to determine one’s actions.	*Psychological development* (8): To promote my personality development. α = 0.82
*Stimulation*: Striving for excitement, novelty, and change.	*Mastering parenting challenges* (6): Parenting is a new social role that I would like to try. α = 0.74
*Hedonism*: Pursuit of pleasure and sensual gratification.	*Enjoying the child* (10): To enjoy touching, holding, and cuddling my child. α = 0.93
***Self-enhancement higher-order values***	***Authority***
*Achievement*: Acquiring personal success through demonstrating competence according to social standards.	*Proving oneself* (5): To prove to myself and others that I can have and raise children. α = 0.79
*Power Dominance*: Aspiration for social status through gaining control and dominance over others.	*Controlling the other* (5): To have somebody who will do everything I tell him/her to do. α = 0.77
*Face*: Obtaining a sense of security and power through maintaining a positive public image and avoiding humiliation.	*Social recognition* (3): To be respected in society as a head of the family. α = 0.83
***Conservation higher-order values***	***Preservation***
*Security Social*: Preserving the wider social structure’s safety, harmony, and stability.	*Strengthening the ingroup* (5): To compensate for the past human losses my people suffered. α = 0.87
*Security Personal*: Preserving safety, harmony, and stability of the self and immediate environment.	*Ensuring support* (6): To have somebody to help me in the future. α = 0.80
*Tradition*: Maintaining and preserving cultural, family, or religious traditions.	*Fulfilling religious obligations* (5): To fulfill god’s commandment to multiply. α = 0.91 *Continuity* (3): To continue the family line. *α* = 0.72
*Conformity*: Limiting actions and urges that might violate rules, laws, social expectations, and norms.	*Yielding to social pressure* (4): To follow the societal norm of having children. α = 0.86
***Self-transcendence higher-order values***	***Perpetuity***
*Benevolence*: Caring for the welfare of others with whom one is in frequent social contact.	*Nurturance* (5): To have somebody to care for. α = 0.79
*Universalism*: Understanding, appreciating, tolerating, and protecting all people’s and nature’s welfare.	*Contribution to humankind* (5): My child will improve the world. α = 0.82

#### Personal value preferences

Value preferences were measured using the Portrait Values Questionnaire, PVQ-57R (Schwartz et al., 2012). For each item, respondents indicate how similar the described person is to them on a 6-point scale, ranging from 1 (*not like me at all*) to 6 (*very much like me*). Item example: “It is important to her/him to avoid upsetting other people” (Conformity). As recommended in previous studies (Czyżkowska and Cieciuch, 2022; Schwartz et al., 2012), participants’ responses were centered on their mean for all values to correct for individual differences in using the response scale. Cronbach’s alphas for the four higher-order values were high: *α* = 0.88–0.93. The higher-order values on the axes’ poles were strongly negatively correlated: *r* = −0.61 for openness to change–conservation, and *r* = −0.56 for self–transcendence–self–enhancement. Therefore, to avoid the problem of multicollinearity, we used axis scores built by subtracting the scores of one pole of an axis from the other. Thus, higher axes scores indicate stronger preferences for openness to change vs. conservation and self-transcendence vs. self-enhancement values.

### Statistical analyses

**Figure 1:**
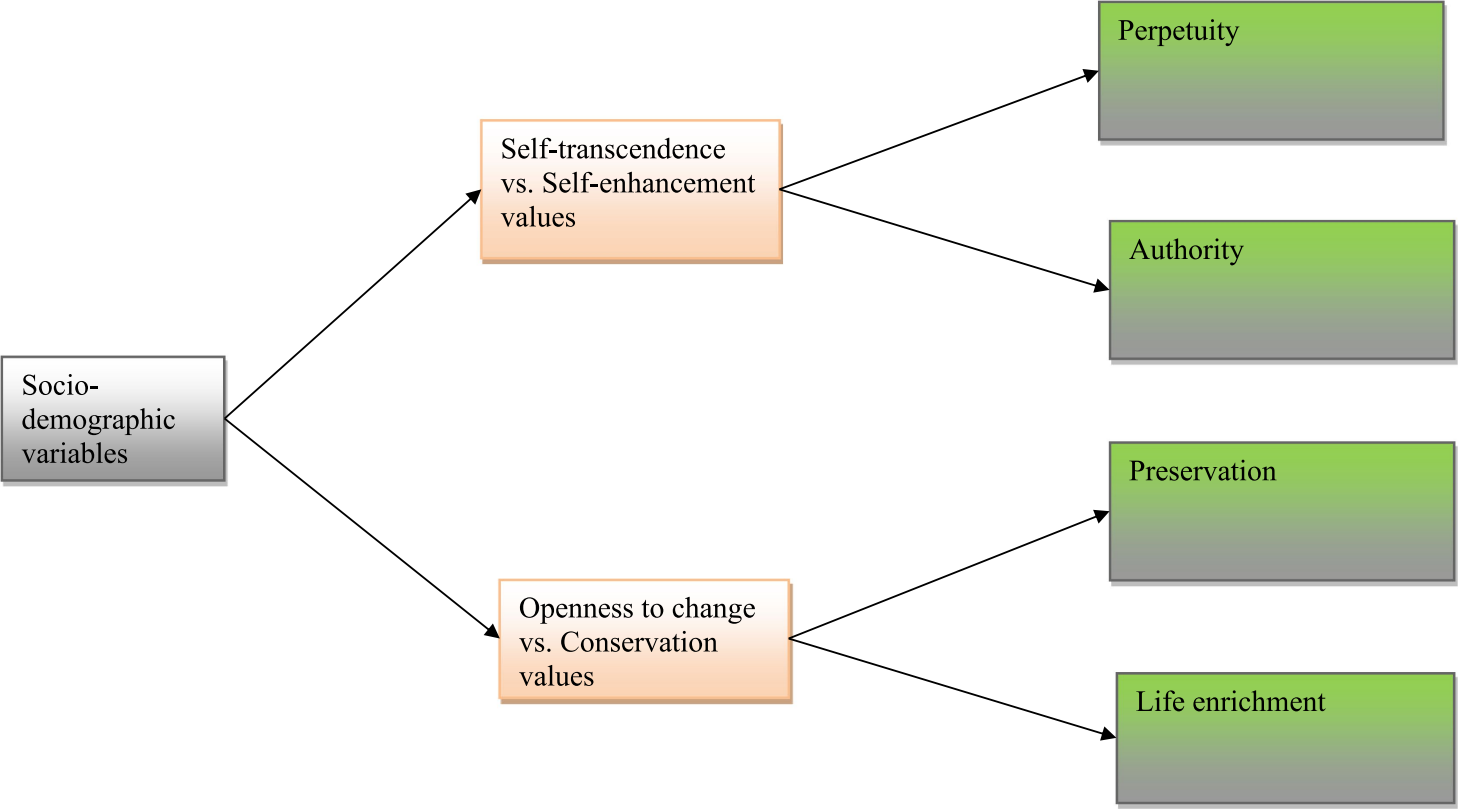
The research model.

We tested the structure of childbearing motivations in two steps. First, using relevant items, we calculated the mean-centered scores for the 13 childbearing motivations. Second, we conducted multidimensional scaling using SPSS for 13 childbearing motivations. After that, we calculated bivariate correlations, means, and standard deviations for all variables in the study: four childbearing clusters, five socio-demographic variables (age, gender, education, income, and religiosity), and two value axes. Finally, we conducted a path analysis using Mplus to test the direct and indirect connections between socio-demographic variables, values, and childbearing motivations (Figure 1 presents the research model). Full information maximum likelihood estimation with robust standard errors was applied to address missing data (Kelloway, 2014; Little and Rubin, 2019). After establishing the model’s goodness of fit, aiming for the most parsimonious model, the model was “trimmed,” i.e., all non-significant paths were excluded (Kelloway, 2014). The direct and indirect effects were tested using the bootstrapping method with 1,000 resamples with a 95% confidence interval.

## Results

### Multidimensional scaling and descriptive statistics

**Figure 2:**
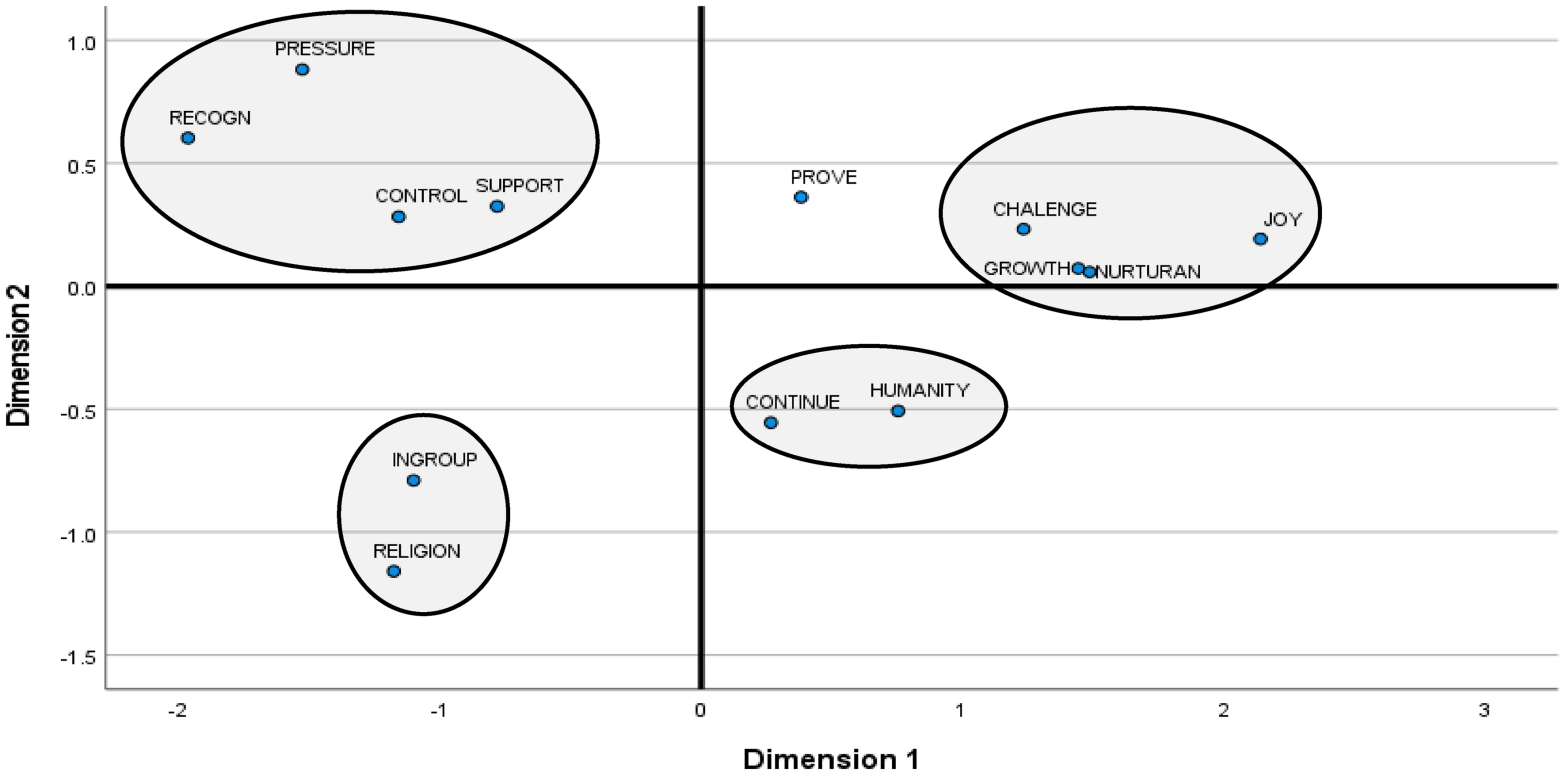
Childbearing motivations (*n* = 13): multidimensional scaling.

Figure 2 presents the results of a Multi-Dimensional Analysis of 13 basic childbearing motivations. The overall quality of the distribution was good (Stress = 0.047; RSQ = 0.990). Four separate clusters were recognizable. *The life enrichment cluster* combined four childbearing motivations: personal growth, mastering the parenting challenges, enjoying the child, and nurturance. *The authority cluster* combined four childbearing motivations: controlling the other, social recognition, yielding to social pressure, and ensuring support. *The preservation cluster* combined strengthening the ingroup and fulfilling religious obligations motivations. Finally, *the perpetuity cluster* combined continuity and contribution to humankind childbearing motivations. Comparing the obtained two-dimensional distribution with the hypothesized structure of childbearing motivations (Table 1), we found that eight basic childbearing motivations fell within their hypothesized clusters, and four fell in a nearby cluster. The motivation to prove oneself fell exactly midway between life enrichment and authority clusters; therefore, it was not included in either of them. This motivation was associated neither with values nor with socio-demographic variables; thus, it was excluded from further consideration. Therefore, the hypothesized structure of childbearing motivations (H1) was corroborated with some corrections.

As predicted, the life enrichment cluster contradicted the preservation cluster (*r* = −0.52, *p* < 0.001), and the authority cluster and perpetuity cluster contradicted each other (*r* = −0.25, *p* < 0.001). However, the life enrichment cluster also contradicted authority (*r* = −0.66, *p* < 0.001) and perpetuity (*r* = −0.28, *p* < 0.001) clusters. Authority and preservation clusters were orthogonal (*r* = 0.00), and the preservation and perpetuity clusters were positively correlated (*r* = 0.28, *p* < 0.001). Thus, the hypothesized pattern of compatibilities and conflicts among childbearing motivations (H2) was corroborated with some corrections.

Table 2 presents the means and standard deviations of the scores for the four motivational clusters. Comparing the four motivational clusters, the most prominent motivation was life enrichment, followed by perpetuity [*t*(1121) = 23.0, *p* < 0.001]. The preservation motivation was less important than perpetuity [*t*(1121) = 31.1, *p* < 0.001], and the authority motivation was less important than preservation [*t*(1121) = 3.12, *p* < 0.001]. The ranking of childbearing motivations was identical for both genders.

**Table 2:** Bivariate correlations, means, and standard deviations.

Variables	Life enrichment	Authority	Preservation	Perpetuity	Gender	Age	Education	Income	Religiosity	SS	OC
Life enrichment	1										
Authority	−0.66^***^	1									
Preservation	−0.52^***^	0.00	1								
Perpetuity	−0.28^***^	−0.25^***^	0.28^***^	1							
Gender	−0.26^***^	0.07^*^	0.16^***^	0.14^***^	1						
Age	−0.01	0.15^***^	−0.10^***^	−0.14^***^	−0.03	1					
Education	0.09^**^	0.09^**^	−0.15^***^	−0.10^***^	−0.19^***^	0.45^***^	1				
Income	−0.01	0.05	−0.06^*^	−0.06^*^	0.10^**^	0.45^***^	0.28^***^	1			
Religiosity	−0.13^***^	−0.21^***^	0.53^***^	−0.17^***^	0.02	0.21^***^	−0.13^***^	−0.09^**^	1		
SS	0.28^***^	−0.33^***^	−0.03	0.08^**^	−0.04	0.04	−0.02	−0.10^***^	−0.01	1	
OC	0.16^***^	0.04	−0.26^***^	−0.09^**^	0.00	−0.02	0.02	0.00	−0.30^***^	−0.09^**^	1
*M(SD)*	0.78 (0.52)	−1.13 (0.78)	−1.01 (1.09)	0.06 (0.77)	1.47 (0.50)	26.2 (4.62)	3.19 (1.20)	2.03 (1.05)	1.89 (0.99)	0.41 (0.48)	0.15 (0.43)

SS – self-transference vs. self-enhancement. OC – openness to change vs. conservation. *p < 0.05, **p < 0.01, ***p < 0.001.

### Path analysis

**Figure 3:**
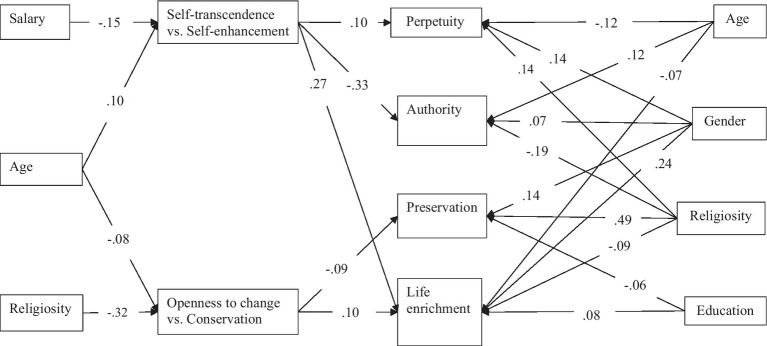
Path analysis: trimmed model (standardized effects).

The goodness-of-fit indexes of a trimmed model were excellent: *χ^2^*(16) = 21.6, *p* = 0.158; *CFI* = 0.998; *TLI* = 0.995; *RMSEA* = 0.018; *SRMR* = 0.015. The proportion of variance explained was significant for all childbearing motivational clusters: preservation (32%), authority (17%), life enrichment (16%), and perpetuity (7%). Figure 3 presents the connections (standardized effects) between variables in the trimmed model. The figure does not include the covariances between the four childbearing motivations and the two value axes to avoid clutter; however, we provide them here. The life enrichment motivation was negatively connected to all other motivations: authority (−0.69), preservation (−0.51), and perpetuity (−0.28). Authority motivation was negatively connected to perpetuity (−0.21) and positively to preservation (0.12). Finally, the connection between preservation and perpetuity motivations was positive (0.21). The two value axes were negatively connected (−0.10).

Confirming the first hypothesis, the connections between values and childbearing motivations were significant and formed the hypothesized pattern. Openness to change vs. conservation values were connected to life enrichment (*β* = 0.10) and preservation (*β* = −0.09). Self-transcendence vs. self-enhancement values were connected to authority (*β* = −0.33), life enrichment (*β* = 0.27), and perpetuity (*β* = 0.10).

Several socio-demographic variables were connected to values. Salary (*β* = −0.15) and age (*β* = 0.10) were connected to self-transcendence vs. self-enhancement, and religiosity (*β* = −0.32) and age (*β* = −0.08) were connected to openness to change vs. conservation. Furthermore, several indirect connections between socio-demographic variables and childbearing motivations were significant. Age was indirectly connected to life enrichment (*β* = 0.019, *p* = 0.047), authority (*β* = −0.034, *p* = 0.003), preservation (*β* = 0.008, *p* = 0.023), and perpetuity (*β* = 0.010, *p* = 0.019); thus, the third hypothesis was corroborated. Religiosity was indirectly connected to life enrichment (*β* = −0.032, *p* < 0.001) and preservation (*β* = 0.029, *p* < 0.001), corroborating the fifth hypothesis. Salary was indirectly connected to life enrichment (*β* = −0.020, *p* < 0.001), authority (*β* = 0.036, *p* < 0.001), and perpetuity (*β* = −0.011, *p* = 0.005); thus, the seventh hypothesis was corroborated. The hypotheses regarding the indirect effects of gender (H4) and education (H6) were not corroborated because these variables were not connected to values.

Finally, socio-demographic variables were directly connected to childbearing motivations. Age was connected to perpetuity (*β* = −0.12), authority (*β* = 0.12), and life enrichment (*β* = −0.07). Gender (1 – *female*, 2 – *male*) was connected to perpetuity (*β* = 0.14), authority (*β* = 0.07), preservation (*β* = 0.14), and life enrichment (*β* = −0.24). Religiosity was connected to perpetuity (*β* = 0.14), authority (*β* = −0.19), preservation (*β* = 0.49), and life enrichment (*β* = −0.09). Finally, education was connected to preservation (*β* = −0.06), and life enrichment (*β* = 0.08).

## Discussion

The present study advances our understanding of childbearing motivations in three ways. First, we revealed the structure of childbearing motivations that reflects their compatibilities and conflicts. Second, we corroborated the connections between context-specific motivational goals, as reflected in childbearing motivations, and general motivational goals, as reflected in personal values. Finally, we demonstrated that socio-demographic differences in childbearing motivations partly result from group differences in values.

### The structure of childbearing motivations

We distinguished 13 childbearing motivations: psychological development, mastering parenting challenges, enjoying the child, proving oneself, controlling the other, social recognition, strengthening the ingroup, ensuring support, fulfilling religious obligations, continuity, yielding to social pressure, nurturance, and contribution to humankind. These childbearing motivations are based on the “child’s values” and are similar to those found in previous studies in the US and Europe (Gormly et al., 1987; Guedes et al., 2015; Hofer et al., 2018; Matias and Fontaine, 2013; Miller, 1994). However, in the present study, we advanced the existing knowledge by theoretically formulating and empirically testing the structure of childbearing motivations, including their compatibilities and conflicts.

We found that childbearing motivations form four clusters, reflecting different goals people strive to achieve by having a child. The first cluster, named *life enrichment*, combines four childbearing motivations: personal growth, challenges of parenting, enjoying the child, and nurturance. This motivational cluster reflects the goals of psychological development, mastering new challenges, developing new capabilities, and enjoying parenting. The next cluster, named *authority*, combines four basic childbearing motivations: controlling the other, social recognition, yielding to social pressure, and ensuring support. This cluster reflects the goal of increasing one’s resources and strengthening one’s position in the societal hierarchy through childbearing. The third cluster, named *preservation*, combines strengthening the ingroup and fulfilling religious obligations. This cluster reflects the childbearing goals aimed at sustaining one’s ethnic and religious group by increasing its membership. Finally, *the perpetuity cluster* combines continuity and contribution to humankind. These childbearing motivations reflect the goal of overcoming human finiteness and providing a sense of eternal existence for oneself and humanity.

The present study revealed a system of affinities and conflicts between the four motivational clusters. As hypothesized, life enrichment contradicted preservation motivations. In addition, life enrichment contradicted the pertinence motivation. These findings highlight the conflict between individualistic and collectivistic motivations for childbearing, as mentioned in previous studies (Inglehart, 2021; Jenkins, 2020). It indicates that people tend to give birth to satisfy either individualistic (life enrichment) or collectivistic (preservation and pertinence) interests. Finally, life enrichment contradicts the authority childbearing motivation. Both these motivations are individualistic; however, the contradiction between them may be explained by applying the distinction between intrinsic and extrinsic motivations (Ryan and Deci, 2017). Life enrichment motivation aims to satisfy the intrinsic goals of self-actualization by obtaining internal rewards in the context of childbearing (the sense of mastery and joy). In contrast, the authority motivation aims to attain extrinsic goals by obtaining social rewards (such as respect, power, and support) through having a child.

Corroborating our hypothesis, authority and perpetuity motivations contradicted each other. This finding further highlights the conflict between individualistic and collectivistic childbearing motivations, as the goals of immediately strengthening one’s position in society through childbirth contradict the goals of symbolically projecting oneself into the future and ensuring a legacy for oneself and humanity (Jenkins, 2020). However, the authority motivation was found to be compatible with the preservation motivation. This finding suggests that hierarchy-related individualistic motivation of strengthening one’s authority through childbirth is compatible with the collectivistic motivation of preservation that prioritizes strengthening one’s group. This finding indicates that the link between collectivism and hierarchy motivations (Gibson, 2010; Singelis et al., 1995) also exists in the context of childbearing.

The comparison among the four motivational clusters revealed that life enrichment is stronger than all other childbearing motivations, and the motivational hierarchy was similar among men and women. This finding suggests that individualistic intrinsic goals, such as self-development and enjoying parenting, are the primary motivations for childbearing among Israeli youths. This finding was unexpected because Israel is a conservative and collectivistic society, compared to other developed countries (Pelham et al., 2022). In addition, this finding raises a question regarding the predominant childbearing motivations in different countries. It is possible that collectivistic and extrinsic motivations for childbearing predominated in previous generations (Jenkins, 2020). However, today, these motivations may be giving way to individualistic and intrinsic motivations (Hashemzadeh et al., 2021; Ranjbar et al., 2024; Ren et al., 2024).

### Connections between childbearing motivations and values

The study results corroborate the hypothesis that context-specific motivations are derived from general motivational goals expressed in personal values. Specifically, life enrichment motivation was associated with high preferences for self-transcendence vs. self-enhancement and openness to change vs. conservation values. It means that people who strive to attain general motivational goals of caring for others, independence, and excitement consider childbearing as a means to attain context-specific goals of self-development, enjoying parenting, and caring for a child. Authority motivation was associated with a high preference for self-enhancement vs. self-transcendence values. It means that people who value achievement and power consider childbearing as a means to promote their social status and increase their social power. Perpetuity motivation was associated with a high preference for self-transcendence vs. self-enhancement values. This suggests that for those who care for others and the universe, giving birth to a child is a means to secure their symbolic immortality and the future of humankind. Finally, preservation motivation was associated with a high preference for conservation vs. openness to change values. It means that people who aim to preserve the status quo give birth to maintain and strengthen their ingroup and religion. Taken together, these results indicate that childbearing motivations are derived from basic motivational goals, as reflected in personal values.

The pattern of connections between childbearing motivations and values helps explain the contradiction between life enrichment and authority childbearing motivations discussed earlier. Specifically, we found that life enrichment motivation is associated with a high preference for self-transcendence values, while authority motivation is related to self-enhancement values, which constitute the opposite poles of the same value axis. In addition, the connections with values help explain the contradiction between life enrichment and preservation motivations, because the first motivation is associated with a high preference for openness to change values, whereas the second motivation is associated with a high preference for conservation values, which constitute the opposite pole of the same axis. It is considered impossible to attain the motivational goals reflected in the opposite poles of a value axis using a single behavior (Sagiv and Roccas, 2021; Schwartz et al., 2012). Thus, the pattern of connections with values explains why life enrichment contradicts the authority and preservation childbearing motivations.

### The effect of socio-demographic variables on childbearing motivations

The results of the present study demonstrate that socio-demographic variables are indirectly connected to childbearing motivations through values. Specifically, salary was connected to self-enhancement values and, through them, associated with stronger authority motivation and weaker life enrichment and perpetuity childbearing motivations. Religiosity was connected to conservation values and, through them, associated with stronger preservation and weaker life enrichment motivations. Finally, age was connected to self-transcendence and conservation values and, through them, associated with stronger life enrichment, perpetuity, and preservation motivations, and weaker authority motivations. These findings indicate that group differences in values may partially explain the socio-demographic differences in childbearing motivations observed in previous studies (Gubernskaya, 2010; Hashemzadeh et al., 2021; Inglehart, 2021; Jenkins, 2020; Mynarska and Rytel, 2022; Ranjbar et al., 2024; Trepczyk and Szablewska, 2024).

Additionally, socio-demographic variables were directly linked to childbearing motivations. Specifically, stronger life enrichment motivation was more characteristic of women, younger individuals, those who are less religious, and more educated people. Preservation motivation was more characteristic of men, as well as more religious and less educated individuals. The authority motivation was found more frequently among men, older individuals, and those who are less religious. Finally, perpetuity motivation was more prevalent among men, younger individuals, and those who are more religious. These results indicate that the predominance of each childbearing motivation is associated with a specific socio-demographic profile. Two mechanisms may be in motion here. First, it may be ideologies, which differ across socio-demographic groups, that prescribe specific childbearing motivations to group members. For instance, conservation ideologies emphasize the preservation and perpetuity motivational goals of childbearing among religious people (Inglehart, 2021; Jenkins, 2020). Patriarchal ideologies stress the importance of authority childbearing motivations for men (Gubernskaya, 2010; Hashemzadeh et al., 2021). Finally, liberal ideologies adopted by many young, educated women emphasize life enrichment motivation (Hashemzadeh et al., 2021; Ranjbar et al., 2024; Ren et al., 2024). The second mechanism directly connecting socio-demographics with childbearing motivations may be modeling, when people belonging to a specific socio-demographic group imitate the cognitive patterns prevalent among group members (Xie et al., 2023; Zentall, 2011).

### Limitations and suggestions for further research

Several study limitations must be considered. First, the study was cross-sectional; therefore, causal inferences cannot be drawn from the results. Future longitudinal research would represent a significant advancement in the current findings. The second limitation relates to the research population. The suggested theoretical model was tested in only one ethno-religious group. Testing the model in other cultural groups and countries is crucial for its generalization. The third limitation relates to the study’s focus on individual-level factors, rather than investigating the mezzo- and macro-level factors (such as parents, peers, and mass media) that might affect childbearing motivations. Fourth, our sample was limited to young adults without children. Further studies should include other age groups and individuals who already have children. Finally, further studies should investigate the motivations of those people who do not want children and decide to remain childfree.

## Conclusion

The present study advances current knowledge in three ways. (1) It corroborates a new comprehensive conceptualization of childbearing motivations as a circumplex consisting of four motivational clusters that parallel the values circumplex. This conceptualization enables us to understand the motivational goals of childbearing and account for the complementarities and conflicts between different childbearing motivations. (2) Our study advances the theory of human values by demonstrating that context-specific motivational goals, in our case, childbearing motivations, are derived from general motivational goals reflected in values. Thus, the research corroborates the existence of a contextualization mechanism that connects general and context-specific motivations. A similar contextualization mechanism has been found in recent studies on romantic relationships (Tartakovsky, 2023b, 2025). Corroborating the existence of the contextualization mechanism also in childbearing may indicate its universality and stimulate research on context-specific motivations in other areas. (3) The research helps us unveil the motivational aspects of socio-demographic variables, demonstrating their direct and indirect effects on childbearing motivations. Thus, differences in childbearing across socio-demographic groups may be partly explained by differences in general and context-specific motivations characterizing these groups.

The present study has practical significance. Corroborating previous studies (Ranjbar et al., 2024; Ren et al., 2024; Trepczyk and Szablewska, 2024), the current study’s results suggest that governmental childbirth-stimulating programs focusing solely on financial support may not be effective. Effective programs must account for childbearing motivations, and first and foremost, for the most prominent motivation of life enrichment. Therefore, government programs must help people to enjoy life and achieve their goals of self-development through parenting. A deeper understanding of childbearing motivations can also be beneficial in reproductive counseling, facilitating informed decisions about family planning. Finally, the study results may help professionals develop motivation-focused interventions facilitating psychological adjustment to normative and challenging reproductive situations.

## Data Availability

The raw data supporting the conclusions of this article will be made available by the authors, without undue reservation.
